# The French general population's attitudes toward lockdown against COVID-19: a fragile consensus

**DOI:** 10.1186/s12889-020-10048-1

**Published:** 2020-12-18

**Authors:** Patrick Peretti-Watel, Pierre Verger, Odile Launay, the COCONEL Study Group, Valérie Seror, Sébastien Cortaredona, Jocelyn Raude, François Beck, Stéphane Legleye, Olivier L’Haridon, Jeremy Ward

**Affiliations:** 1 Aix Marseille Université, IRD, AP-HM, SSA, VITROME, IHU Méditerranée Infection, 19-21 bd Jean Moulin, 13005 Marseille, France; 2 Southeastern Health Regional Observatory (ORS Paca), Marseille, France; 3 Inserm CIC 1417, Faculté de Médecine Paris Descartes, Univ Paris, AP-HP, Hôpital Cochin, Paris, France

**Keywords:** COVID− 19, Lockdown, France, Socioeconomic factors

## Abstract

**Background:**

In March 2020, as the coronavirus disease 2019 (COVID− 19) pandemic was spreading across the globe, many countries have implemented unprecedented lockdown measures. But how populations did react to these measures? We examined the case of France. Our aims were threefold: assessing some aspects of their impact on French’s daily living conditions; investigating their attitudes toward the lockdown; investigating the factors associated with these attitudes.

**Methods:**

A cross-sectional online survey was carried out 10 days after the nationwide lockdown (from March 27th to March 29th 2020), among a representative sample of the mainland French population aged 18 and over. A quota sampling method was applied to achieve a sample of 1012 respondents. We used a cluster analysis to obtain contrasted attitudinal profiles, and logistic regressions to investigated which factors were associated to these profiles.

**Results:**

After 10 days of lockdown, there were already significant consequences regarding respondents’ living conditions and mental health. Most respondents supported the current lockdown. However, it appeared as a stopgap measure due to a lack of alternatives, and a large majority acknowledged its heavy drawbacks. We found three contrasted attitudinal profiles: *full support* (38%), *strong but critical support* (31%), *limited support* (31%). Regarding respondents’ SES, low-income and low-education respondents were more likely to display critical or limited support to the lockdown, as well as those who reported deteriorated living conditions or psychological distress.

**Conclusions:**

In France, the large public support to the lockdown was fragile. First, it was a critical consensus anchored in current controversies and recent social struggles. Second, it was weaker among people with a lows SES, especially since the lockdown have exacerbated preexisting social inequalities.

## Background

In March 2020, as the coronavirus disease 2019 (COVID− 19) pandemic was spreading across the globe, an increasing number of countries implemented unprecedented mitigation measures in order to slow it down [1, 2]. As a result, 1 month later billions of people worldwide were living under lockdown measures. Europe was declared the “epicenter” of the global COVID− 19 pandemic by the World Health Organisation on March 13th [3], and many European countries opted for restrictive mass quarantines, especially in the five countries which reported the highest COVID− 19 death tolls: Italy, Spain, France, the United Kingdom and Belgium [4]. For public health decision-makers, this strategy has added one more type of uncertainty to an already long list of crucial unknowns (e.g. clinical severity, extent of transmission and infection, treatment options [1, 5]): how would the public react to such stringent measures across different sociocultural contexts? [6].

Previous studies suggest that, in a pandemic context, populations would be likely to comply with mitigation measures but would be challenged to do so if their income or job were threatened, and also needed to be reminded about the collective benefits of quarantine measures [7, 8]. Such studies also highlighted the psychological impact of quarantine, which was confirmed recently by a nationwide study conducted in China on February 2020 [8, 9].

In the present study, we would like to address this issue by examining the case of France. During the last decade, the relationship between the French public and health authorities has become complicated, especially when it comes to prevention and the degree of coercion that could be deemed socially acceptable [10]. A decade ago, French public health authorities embarked in a mass vaccination campaign against pandemic 2009 A/H1N1 influenza and were widely criticized for “over-reacting” [11]. The vaccination campaign was not only a complete failure -only 8% of French people got vaccinated [12] – but it also ushered in a conflictual era for epidemic risk management.

Using a nationally representative online survey conducted 10 days after the nationwide lockdown was introduced (March 17th 2020), our aims were threefold: assessing the impact of the lockdown on French people’s daily life, including their psychological well-being; investigating the French public’s attitude toward the nationwide lockdown in order to identify some contrasted profiles; investigating the factors associated with these profiles (including their social differentiation).

## Methods

### Design and sample

A cross-sectional online survey was carried out during the lockdown in France, between 27th and 29th of March 2020 among a representative sample of the mainland French population aged 18 and over. This sample was randomly selected from an online research panel of more than 750,000 nationally representative households of the French general population developed and maintained by IFOP (Paris, France), a survey research firm (https://www.ifop.com/). A quota sampling method was applied to achieve a sample of 1000 respondents, representative for the French adult population in terms of age, gender, occupation and population in the area of residence. The sample size of 1000 adults was calculated to obtain a maximum margin of sampling error of ±3.1 percentage points for an estimated proportion of 50% (final sample size: 1012 people aged 18 and over). A total of 25,800 households were randomly drawn to reach the sample size within 3 days. To be eligible, panelists had to be aged over 18 years and having not answered a previous survey wave on COVID− 19. Prior information on the panelists was used to determine eligibility and to draw a stratified random sample with oversampling of panelists with low response rates (e.g. panelists aged 18–24, blue-collar workers). To limit coverage bias, due to the fact that not all people use the internet, and, among users, that not all of them are willing to participate in web surveys, random sampling was stratified to match French official census statistics for gender, age, occupation (8 categories), population in the area of residence (5 categories) and region (12 categories). Participants gave their consent electronically. The study design was approved by the ethical committee of the University Hospital Institute Méditerranée Infection (#2020–018).

### Data collected

In addition to background socio-economic variables (gender, age, educational level), we computed each respondent’s equivalized household income per month, taking the size and composition of the household into account [13], and using the Organization for Economic Cooperation and Development’s scale. Then we built a three-item indicator: ‘Low income’ refers to the first quartile of the equivalized household income, ‘medium income’ to the second and third quartiles, ‘high income’ to the last quartile.

Regarding potential consequences of the lockdown on daily life, we collected information about self-reported financial difficulties experienced because of it, as well as the current occupation status: still working out of home, teleworking, unemployed due to the lockdown, unemployed or out of work before the lockdown. Respondents were also asked about their household size and housing surface area to identify those who were confined in an overcrowded household (< 194 square feet-per-capita). Moreover, we used the Mental Health Inventory (MHI-5), which is a brief, valid, and reliable international instrument for assessing mental health in adults [14]. Composed by five items, the MHI-5 is a subscale of the SF-36 health-related quality of life measure. Several studies have provided evidence that the MHI-5 is a reliable instrument to assess depression and anxiety in the general population, including France (e.g., [15–17]). We used a cut-off score of 56 as an indicator of psychological distress [18]. Respondents were also asked about confirmed cases of COVID− 19 in their household or among their relatives (family, friends), as it may impact their attitudes toward the lockdown.

Finally, the questionnaire investigated respondents’ level of agreement with twelve statements relating to various aspects of the current lockdown, including its necessity, effectiveness, drawbacks, alternative measures, and whether it should be strengthened or softened, using a four-point Likert scale, from “strongly agree” to “strongly disagree”) (see Table 1).

**Table 1:** Some impacts of the lockdown and the COVID-19 epidemic on French’s daily life, March 27–29, COCONEL Survey (*N* = 1012)

	column %
Experiencing financial difficulties due to the lockdown:	
-no	81%
-yes	19%
Current occupation status:	
-still working out of home	20%
-teleworking	11%
-unemployed due to the lockdown	21%
-unemployed or out of work before the lockdown	48%
Confined in an overcrowded housing:	
-no	91%
-yes	9%
Mental health (MHI-5):	
-no psychological distress	64%
-psychological distress	36%
Cases of COVID-19 in the household/among relatives:	
-no	89%
-yes	11%

In our questionnaire, 18 questions dealt with the living conditions of the respondents, 8 questions dealt with health status (including MHI-5), 6 questions dealt with isolation and socialization, and 14 questions investigated respondents’ attitudes toward the lockdown. The whole questionnaire (in French) is available on request.

### Statistical analysis

Sampling weights were applied to all statistics to produce the proper representation. We first considered the level of agreement with the lockdown-related statements (recoded into binary outcomes: agree vs disagree) and their judgment on the issues listed above.

Second, we performed a cluster analysis on the twelve lockdown-related statements, in order to summarize the variety of respondents’ answers in a few contrasted profiles, and to detect meaningful patterns of attitudes expressed toward the current lockdown. Items measuring agreement were encoded from 1 (“strongly disagree”) to 4 (strongly agree”). These scores were transformed into Z-score form prior to clustering with the usual agglomerative hierarchical procedure [19]. We opted for the three-cluster solution. To describe the distribution of participants’ answers to the 12 items according to the clusters, we used the binary recoding (agree/disagree) and χ^2^ tests.

Third, we used bivariate analyses and a multiple logistic regression to investigate factors associated with these profiles. We used a multinomial logistic model which consists in taking one modality as the reference, then to compare each separately to this reference [20]. We compared Clusters 1 & 2, then Clusters 1 & 3, using background variables and potential consequences of the current situation as candidate covariates, with a stepwise selection method (entry threshold *p* = 0.05).

## Results

A total of 1012 adults over 18 completed the online survey between March 27th and March 29th 2020 (completion rate 72%). No differences for the socio-demographic and geographic variables used for stratification were found between respondents and the French general population as observed in the latest census statistics. The mean age of respondents was 49.9 years old (min = 18, max = 99), 47.6% were men and 18.2% had a high educational level (> 2 years completed at university).

Regarding attitudes toward the lockdown, most respondents supported it (see Table 2). Reciprocally, only one out of five considered that the lockdown is disproportionate considering the real severity of the epidemic, or agreed that it should be softened to be more bearable. The lockdown however appeared as a stopgap measure due to a lack of alternatives (because of the lack of hospital resources, masks and screening tests). On the one hand, a large majority acknowledged its heavy drawbacks, and four out of ten considered it implied too much restriction on civil liberties. On the other hand, most respondents agreed that it could be an opportunity to develop local solidarity.

**Table 2:** French’ s attitudes toward the lockdown, cluster analysis, March 27–29, COCONEL Survey (*N* = 1012)

Agreement with the following statements (column %):	Total sample	Cluster 1 38%	Cluster 2 31%	Cluster 3 31%
The lockdown …	(column %)			
… should last several more weeks to be effective	94%	100%	99%	80% *b*
… has already disastrous economic consequences	93%	88% *b*	99% #	93%
… is an opportunity to develop local solidarity	91%	91%	98% #	84% *b*
… is the only effective way to fight the epidemic	89%	98% #	97% #	69% *b*
… should be strengthened to be effective	80%	87%	96% #	56% *b*
… will cause family tragedies	76%	67% *b*	86% #	79%
… should be less coercive to be more acceptable	22%	4% *b*	19%	47% #
… is disproportionate considering the real severity of the epidemic	20%	1% *b*	21%	41% #
… is the consequence of the lack of hospital resources	66%	39% *b*	90% #	75%
… could be replaced by mass screening tests	65%	39% *b*	87% #	76%
… could have been avoided by the widespread wearing of masks	50%	23% *b*	76% #	59%
… causes too much restriction on civil liberties	41%	10% *b*	52%	69% #

# proportion is significantly higher (*p* < 0.05) than in the rest of the sample

*b* proportion is significantly lower (*p* < 0.05) than in the rest of the sample

### Some impacts of the current situation on respondents’ daily life

After 10 days of lockdown, 19% of respondents declared that they were already experiencing financial difficulties due to its consequences, and 21% were unemployed because of it. The proportion of those who were still working out of their homes (20%) was quite double to those who had the opportunity of teleworking (11%) (see Table 1). Regarding housing conditions, 9% of participants were confined in an overcrowded household. One out of three (36%) displayed psychological distress according to the MHI-5 score. A limited part of the sample (11%) reported knowing someone infected by COVID− 19 in their household, or among their family and friends.

### Patterns of attitudes toward the lockdown

Regarding the results of the cluster analysis, first there was consensus on three assertions, as they were supported by at least 80% of participants across each cluster: the current lockdown should last several more weeks to be effective, it has already disastrous economic consequences, and it is an opportunity to develop local solidarity.

The largest group, Cluster 1, gathered 38% of participants. Almost all in this group agreed that the lockdown is the only effective way to fight the epidemic (98%) and that it should be strengthened to be effective (87%), and 67% of them also considered it will cause family tragedies. A majority of them disagreed with the other opinions. Thus we labelled this profile *full support* to lockdown.

Respondents in Cluster 2 (31% of the sample) shared similar views, as almost all of them supported the lockdown and its strengthening. Nevertheless, they were also more prone to emphasize its drawbacks: 99% agreed it has already disastrous economic consequences, 86% agreed it will cause family tragedies and 52% considered it restricts civil liberties too much. In addition to these critical views, three other opinions were supported by a large majority of them: the lockdown is the consequence of the lack of hospital resources (90%), it could be replaced by mass screening tests (87%), it could have been avoided by the widespread wearing of masks (76%). These opinions were not contradictory with their current support of the lockdown: at the time of this survey, there was a face masks shortage, and mass screening tests were not available at large scale. This profile could be summarized as *strong but critical support*.

Cluster 3 also gathered 31% of the sample. Among them, “only” 69% agreed that the lockdown is the only effective way to fight the epidemic, and 56% that it should be strengthened to be effective. This weaker support of the lockdown was also illustrated by their higher level of agreement with two other statements: the lockdown should be less coercive to be more acceptable (47% agreed); it is disproportionate considering the real severity of the epidemic (41% agreed). Regarding drawbacks of the current lockdown, or alternatives to it, members of Cluster 3 were much more critical than those of Cluster 1, but not as much as those of Cluster 2, except that 69% of them stated that the current lockdown causes too much restriction on civil liberties. Thus these responses expressed a *limited support* of the lockdown.

### Factors associated with attitudinal patterns

In bivariate analyses, attitudes toward the lockdown were very similar for men and women, while younger respondents were more likely to express *limited support*: 48% of respondents aged 18–25 belonged to Cluster 3, vs 24 to 32% of other age groups (see Table 3). Regarding educational level, the more educated displayed more frequently a *full support* of the lockdown (51% among those who completed more than 2 years at university, vs 32% among those who did not complete high-school). Regarding household income level, 55% of high-income respondents expressed *full support* of the lockdown (vs only 18% among low-income respondents). Conversely, low-income respondents were more likely to express either *strong but critical* (37%) or *limited support* of the lockdown (45%).

**Table 3:** Factors associated with French’s attitudes toward the COVID-19 lockdown, logistic regressions, March 27–29, COCONEL Survey (*N* = 1012)

	Cluster 1 38%	Cluster 2 31%	Cluster 3 31%	Cluster 2 vs Cluster 1	Cluster 3 vs Cluster 1
	*row%*			*adjusted odds ratios*	
Gender					
- man (*n* = 482)	40%	28%	32%	NS	NS
- woman (*n* = 530) (ref.)	36%	33%	31% ns		
Age (in years):					
- 18–25 (*n* = 113)	31%	21%	48%	0.76 ns	NS
- 26–45 (*n* = 318)	41%	27%	32%	0.86 ns	
- 46–65 (*n* = 339) (ref.)	38%	32%	30%	-1-	
- > 65 (*n* = 242)	39%	37%	24%***	1.75**	
Educational level:					NS
- < High-school (*n* = 515) (ref.)	32%	38%	30%	-1-	
- High-school, 1st university degree (*n* = 313)	40%	28%	32%	0.75 ns	
- > 2 years completed at university (*n* = 184)	51%	15%	34%***	0.39***	
Household’s income level:					
- low income (<Q1, *n* = 216)	18%	37%	45%	2.25**	2.46***
- medium income (Q1-Q3, *n* = 566) (ref.)	39%	31%	30%	-1-	-1-
- high income (>Q3, *n* = 230)	55%	24%	21%***	0.74 ns	0.58**
Experiencing financial difficulties due to the lockdown					
-no (*n* = 822) (ref.)	43%	29%	28%	-1-	-1-
-yes (*n* = 190)	19%	38%	43%***	2.40***	2.16**
Current occupation status:					
-still working out of home (*n* = 203) (ref.)	38%	29%	33%	NS	NS
-teleworking (*n* = 107)	58%	14%	28%		
-unemployed due to the lockdown (*n* = 212)	36%	33%	31%		
-unemployed/out of work before the lockdown (*n* = 490) (ref.)	35%	34%	31%***		
Confined in an overcrowded housing:					
- no (*n* = 918) (ref.)	39%	31%	30%	NS	NS
- yes (*n* = 94)	25%	29%	46%**		
Mental health (MHI-5)					
- no psychological distress (< 56, *n* = 644) (ref.)	44%	28%	28%	-1-	-1-
- psychological distress (≥56, *n* = 368)	29%	35%	36%***	1.61**	1.52*
Cases of COVID-19 in the household/among relatives					
- no (*n* = 898) (ref.)	38%	31%	31%	NS	NS
- yes (*n* = 114)	40%	27%	33% ns		

***,**,*, ns: respectively significant at *p* < 0.001, *p* < 0.01, *p* < 0.05, non significant (χ^2^ independence test for bivariate analyses, Wald’s χ^2^ for adjusted odds ratios)

NS: variable not selected by the stepwise selection procedure (entry threshold *p* < 0.05)

Regarding respondents’ current situation, those who were already experiencing financial difficulties due to the lockdown were much less likely to fully support the measure (only 19% belonged to Cluster 1), as well as those who displayed psychological distress (29% belonged to Cluster 1 compared to 44% in those without psychological distress). *Full support* of the lockdown was more frequent among respondents who were currently teleworking (58%) than in the other occupational status categories, while those confined in an overcrowded housing were more likely to express *strong but critical support* (46%) than the participants not living in such conditions. Finally, attitudes toward the lockdown did not differ according to the personal proximity of the disease (knowledge of potential cases of COVID-19 in the household or among relatives).

In multivariate analyses, we first compared Cluster 2 & 1. Once controlled for other selected covariates, respondents aged over 65 were more likely than their younger counterparts to express *strong but critical support* instead of *full support* (adjusted odds ratio (aOR) = 1.75), while the most educated were less likely to do so (aOR = 0.39). *Strong but critical support* was also more likely among low-income respondents (aOR = 2.25), those experiencing financial difficulties due to the lockdown (aOR = 2.40) and those displaying psychological distress (1.61).

Finally, we compared the *limited support* profile to the *full support* one. Three differentiating factors remained statistically significant in multivariate analysis. Respondents who experienced financial difficulties due to the lockdown were more likely to express *limited support* (aOR = 2.16), as well as those displaying psychological distress (1.52). The results were dramatically contrasted when considering household income level, as adjusted odds ratios were statistically significant for both low-income and high-income respondents when compared to medium-income (respectively aOR = 2.46 and aOR = 0.58).

## Discussion

### A critical consensus anchored in current controversies and recent social struggles

According to our results, after 10 days of implementation, a strong consensus had been maintained in the French general population in favor of the national lockdown. This is consistent with recent studies conducted in various sociocultural contexts, including European countries, which found overall considerable public support for lockdown measures [21–23], despite awareness of economic and social consequences [24]. One of them also found that such support was stronger in southern Europa (France, Portugal, Italy), despite the fact that trust in authorities was lowest there, especially in France [23]. We found indeed that, in France, the consensus remained fragile. First, agreement with the lockdown was often associated with critical views toward the public health strategy of French authorities: for a majority of respondents, the lockdown appeared as a stopgap measure due to lack of alternatives. These attitudes strongly echoed vivid controversies that grew in the media since the beginning of the lockdown: public health authorities have been strongly criticized for the lack of testing capacities, and because they did not replenish their stocks of face masks (10 years ago France had stocks of one and a half billion masks, but only 150 million remained at the outbreak of the epidemic). Beyond these controversies directly related to the current health crisis, our results also echo social conflicts which occurred in 2018–2019: regarding the lack of hospital resources, French hospital workers had been on strike for months during the previous year claiming more resources; and regarding restriction on civil liberties, many opponents accused the government of impinging on civil liberties during the so-called ‘yellow vests’ protest movement that started in the fall of 2018.

### Socioeconomic status and support to the lockdown

Moreover, the consensus in favor of the national lockdown has been, since the start, socially differentiated. Regarding respondents’ SES, we found that low-income and low-education respondents were more likely to display critical or limited support of the lockdown. This is not surprising, as usually a high SES is associated with a greater trust in public health authorities, as illustrated by public reactions during the 2009 A/H1N1 flu pandemic: both intention to be vaccinated against 2009 A/H1N1 virus and actual vaccine uptake were reported to be positively correlated with educational and income levels and higher professional and managerial occupation [3, 25–28].

In the present case, however, this relationship was amplified by the fact that the lockdown differentially impacted daily living conditions, and exacerbated preexisting social inequalities. Indeed, experiencing financial difficulties due to the lockdown, being unemployed because of it, being confined in overcrowded conditions were correlated to critical or limited support. Indeed, because these daily life experiences were strongly correlated to respondents’ SES [29], most of the corresponding statistical effects became non-significant in multivariate analyses. This socially differentiated interpretation could be extended to mental health. Of course, we did not have any assessment of our individual respondents’ mental health prior to the lockdown. But a national representative survey conducted in 2017 with the MHI-5 in the French general adult population provides a reference point: at the time, 27% of French adults could be considered in psychological distress [30]. As could be expected [8, 9, 24], our results suggest that the psycho-social consequences of the lockdown are likely to have deteriorated mental health at a populational level: among our respondents the prevalence of psychological distress reached 36% (vs 27% among French adults in 2017), and, unsurprisingly, this prevalence was higher among low-income people [30]. Although our data did not allow us to estimate the specific impact of the lockdown and therefore uncertainty remains about the respective role of preexisting conditions versus direct psychological consequences of the health crisis, multivariate analysis in our sample clearly establishes that poor mental health was a significant correlate of being more uneasy with the public health response to the COVID-19 pandemic (critical or limited support to the lockdown). This is consistent with recent studies which found that, during the lockdown, distrust toward government action was negatively correlated to psychological well-being [24, 31].

### Limits of the study

We have to acknowledge several limitations of the present study. The Covid-19 crisis is affecting data collection activities and online surveys are an effective way to carry out surveys despite the lockdown. However, online data collection has some well-established drawbacks that we have tried to minimize. The first issue relates to unequal access to the Internet. The Internet coverage is quite high among French households (estimated at 89% in 2018 [32]). Moreover, random sampling in our survey was stratified to match French official census statistics for gender, age, occupation and population in the area of residence and region. Second, a selection bias can exist. In order to limit this risk, the invitational email did not mention the theme of the survey. Third, online surveys share with other survey methods the general limitations of results based on respondent’s self-report, especially social desirability bias. Nevertheless, self-administered questionnaires tend to yield fewer reports in the socially desirable direction than do interviewer-administered questionnaires, and online surveys may have the lowest social desirability bias [33].

## Conclusions

As long as strategies for mitigation of the COVID-19 crisis rely on massive and rapid behavior change, it is crucial to monitor public perceptions and reactions [34]. Among the French population, according to our representative study, the consensus in favor of the national lockdown was fragile, first because it was anchored in current controversies and recent social struggles, secondly because of its social differentiation. Regarding attitudes toward public policies, such social differentiation was far from unusual, but in the present case it was magnified by the deleterious consequences of the lockdown which heavily contributed to increase preexisting social (health) inequalities.

Further research should address lay people’s attitudes toward lockdown and other mitigation measures, their social differentiation and their relationship with psychological well-being, including with a dynamic perspective, as they may change over time. Taking into account social differences in the population’s reactions will be absolutely key in both the short and mid-term future, as public health policy has to shift to other strategies rather than national lockdowns to control the pandemic. Moreover, the unprecedented economic recession resulting from the lockdown will have major consequences on the population’s health with an expected higher increased morbidity and mortality in the most vulnerable groups [35, 36]. Explicitly dealing with health and social inequalities should therefore be put at the core of all national agendas.

## Supplementary Information

**Additional file 1:.** COCONEL Survey, 1st Wave.

## Data Availability

The datasets used and/or analysed during the current study are available from the corresponding author on reasonable request.
