# Supplementary material for: The French general population's attitudes toward lockdown against COVID-19: a fragile consensus
Source: BMC Public Health. 2020 Dec 18;20:1920. doi: 10.1186/s12889-020-10048-1 (PMC7746918; doi:10.1186/s12889-020-10048-1)
Supplement: Supplementary file 1 — Additional file 1:. COCONEL Survey, 1st Wave. [file 12889_2020_10048_MOESM1_ESM.pdf]

March, 26th, 2020

COCONEL Survey, 1st Wave.

A. Living & housing conditions.

Q1. How do you pull through with you household's income?
It's very hard
It's hard
Easily
Very easily

If 'very hard' or 'hard'

Q2. Is it because income losses due to the lockdown?
Strongly agree
Somewhat agree
Somewhat disagree
Strongly disagree

For economically active people

Q3. Are you currently still working?
1 Yes, full-time work outside home
1 Yes, part-time work outside home
2 Yes, I am teleworking
3 No, I stopped working due to the lockdown
4 No, I am looking for a job

Q4. There are currently X people living in your household. How old are they?

Q5. Are you currently living……?
1 In your usual home
2 In your second home
3 At a relative's place
4. At a friend's place
5. Other (specify)

Q6. And the place where you are currently living is…?
1 An individual house
2 An apartment

3 An independent room with its own entrance
4 Other (specify)

Q7. What is approximately the total surface area of this place (in square meters)?

Q8. How many rooms are there in this place?

Q9. In this place, is there…? (Yes/No)
1 A balcony
2 A terrace
3 A garden or a plot of land
4 A courtyard

For each 'Yes' response to Q9

Q10. Do you share this balcony / terrace… with other households?
- Yes
- No

B. Health Status

Q11. Generally speaking, how is your health now?
1 Very good
2 Rather good
3 Rather bad
4 Very bad

Q12. Are you currently living with a chronic condition which requires medical care (example: diabetes, cardiac or respiratory disease…)?
Instructions: do not take into account temporary conditions, such as flu.
1 Yes. Wich one(s)? (specify))
2 No
3 I don't know

Q13 Last winter (2019-2020), did you get vaccinated against the seasonal flu?
Yes/No

Q14/15.During the past month, how much of the time…: ***(one response per line)***

	All of the time	Most of the time	A good bit of	Some of the time	A little of the time	None of the time

			the time			
…have you been a very nervous person?	☐1	☐2	☐3	☐4	☐5	☐6
…have you felt so down in the dumps that nothing could cheer you up?	☐1	☐2	☐3	☐4	☐5	☐6
…have you felt calm and peaceful?	☐1	☐2	☐3	☐4	☐5	☐6
…have you felt downhearted and blue?	☐1	☐2	☐3	☐4	☐5	☐6
…were you a happy person?	☐1	☐2	☐3	☐4	☐5	☐6

Q16. How do you describe your present condition?
1 I am / have been infected with the novel coronavirus (medically confirmed)
2 I have symptoms of it, but it is not confirmed
3 I have no symptom, but I have been in contact with an infected person
4 I have no symptom and I don't think I have been infected

Q17. And within your household, is there a confirmed case of the novel coronavirus?
1 No
2 No, but someone has symptoms
3 No, but someone has been in contact with an infected person
4 Yes, someone has been tested positive to the novel coronavirus

Q18. Among your relatives (family, friends), has some vbeen infected by the novel coronavirus?
1 Yes, several of them have been tested positive
2 Yes, one of them have been tested positive
3 No, but a least one has symmptoms
4 No

C. Socialisation & isolation

Q19. The day before, how many times did you get out of your housing?
Instruction: going to the garden or on the balcony does not count…

- I stayed at home
- once
- twice
- more often

If at least 'once'

Q20. The day before, how much time (approximately) did you spend out of your housing? (specify how many hours and minutes)

If at least 'once'

Q21. For which motives did you get out (several responses available): (random order)
- For work
- For shopping
- For a walk
- For doing sports
- To help a kin/a friend
- To go to the pharmacy
- To go to a medical appointment

Q22. And the day before, did a person from outside your household visit you?
- Yes, one visit
- Yes, several visits
- No

Q23. The day before, how much time did you spent on your phone (SMS do not count)?
Q24. And how many people did you talk to?

Q25. The day before, how much time did you spent chatting with other people on the social media (SMS, Insta, Whatsapp…)?
Q26. And how many people did you chat with?

D. Perceptions of the Covid-19

Q27. If a vaccine becomes available, would you get it?
- Yes, certainly
- Yes, probably
- Probably not
- Certainly Not

Q28-30. The following statements deal with the current lockdown. Please now give your opinion on each of them (random order).
The lockdown…
…is the only effective way to fight the epidemic
…is disproportionate considering the real severity of the epidemic
…causes too much restriction on civil liberties
…should last several more weeks to be effective
…should be strengthened to be effective
…should be less coercive to be more acceptable
…will cause family tragedies
…has already disastrous economic consequences
…is the consequence of the lack of hospital resources
…could have been avoided by the widespread wearing of masks
…could be replaced by mass screening tests
…is an opportunity to develop local solidarity

Strongly agree / somewhat agree / somewhat disagree / strongly disagree

Q31. According to you, when will the covid-19 pandemic end?

___ months & ___ weeks
